# Towards universal health coverage in Vietnam: a mixed-method case study of enrolling people with tuberculosis into social health insurance

**DOI:** 10.1186/s12961-024-01132-8

**Published:** 2024-04-02

**Authors:** Rachel Forse, Clara Akie Yoshino, Thanh Thi Nguyen, Thi Hoang Yen Phan, Luan N. Q. Vo, Andrew J. Codlin, Lan Nguyen, Chi Hoang, Lopa Basu, Minh Pham, Hoa Binh Nguyen, Luong Van Dinh, Maxine Caws, Tom Wingfield, Knut Lönnroth, Kristi Sidney-Annerstedt

**Affiliations:** 1 Friends for International TB Relief, Hanoi, Vietnam; 2 Department of Global Public Health, WHO Collaboration Centre on Tuberculosis and Social Medicine, Karolinska Institutet, Stockholm, Sweden; 3 Centre for Development of Community Health Initiatives, Hanoi, Vietnam; 4 IRD VN, Ho Chi Minh City, Vietnam; 5 USAID Vietnam, Hanoi, Vietnam; 6 National Lung Hospital, Hanoi, Vietnam; 7 Centre for TB Research, Department of Clinical Sciences, Liverpool School of Tropical Medicine, Liverpool, UK; 8 Birat Nepal Medical Trust, Kathmandu, Nepal

**Keywords:** Social health insurance, Tuberculosis, Vietnam, Universal health coverage, Health financing transition, Financial protection

## Abstract

**Background:**

Vietnam’s primary mechanism of achieving sustainable funding for universal health coverage (UHC) and financial protection has been through its social health insurance (SHI) scheme. Steady progress towards access has been made and by 2020, over 90% of the population were enrolled in SHI. In 2022, as part of a larger transition towards the increased domestic financing of healthcare, tuberculosis (TB) services were integrated into SHI. This change required people with TB to use SHI for treatment at district-level facilities or to pay out of pocket for services. This study was conducted in preparation for this transition. It aimed to understand more about uninsured people with TB, assess the feasibility of enrolling them into SHI, and identify the barriers they faced in this process.

**Methods:**

A mixed-method case study was conducted using a convergent parallel design between November 2018 and January 2022 in ten districts of Hanoi and Ho Chi Minh City, Vietnam. Quantitative data were collected through a pilot intervention that aimed to facilitate SHI enrollment for uninsured individuals with TB. Descriptive statistics were calculated. Qualitative interviews were conducted with 34 participants, who were purposively sampled for maximum variation. Qualitative data were analyzed through an inductive approach and themes were identified through framework analysis. Quantitative and qualitative data sources were triangulated.

**Results:**

We attempted to enroll 115 uninsured people with TB into SHI; 76.5% were able to enroll. On average, it took 34.5 days to obtain a SHI card and it cost USD 66 per household. The themes indicated that a lack of knowledge, high costs for annual premiums, and the household-based registration requirement were barriers to SHI enrollment. Participants indicated that alternative enrolment mechanisms and greater procedural flexibility, particularly for undocumented people, is required to achieve full population coverage with SHI in urban centers.

**Conclusions:**

Significant addressable barriers to SHI enrolment for people affected by TB were identified. A quarter of individuals remained unable to enroll after receiving enhanced support due to lack of required documentation. The experience gained during this health financing transition is relevant for other middle-income countries as they address the provision of financial protection for the treatment of infectious diseases.

**Supplementary Information:**

The online version contains supplementary material available at 10.1186/s12961-024-01132-8.

## Background

Contributing to universal health coverage (UHC) by improving access to fair and sustainable health financing, of which one mechanism is health insurance, has become a priority among low- and middle-income countries [1, 2]. Many countries in the Asia Pacific region have made steady progress towards UHC coverage through sustained political commitments and fiscal policy aligned with their commitment [3]. By 2020, 27 countries had implemented a social health insurance (SHI) financing mechanism, which typically includes open enrollment for the full population along with partial or full subsidization of healthcare costs for vulnerable groups [4].

Vietnam’s first SHI scheme was piloted in 1989 and grew through successive pilots and expansions. In 2009 the national-level Health Insurance Law (HIL) went into effect, uniting the existing health insurance programs and schemes for the poor [5]. Amendments to the HIL effective in 2015 made SHI compulsory for all and pooled risk by re-structuring registration around the household unit [4]. A household in Vietnam is defined by inclusion in the ‘family book*’,* the national system of family and address registration [6].

Access to SHI in Vietnam increased rapidly, principally through subsidization of premiums. Specific groups were enrolled automatically with full subsidy, including vulnerable populations (e.g., households classified as ‘poor’, children aged < 6, people aged > 80), pensioners and meritorious groups (e.g., veterans). Partial premium subsidization was also available for students, households classified as ‘near-poor’ and some farmers [7]. More than half of SHI members are entitled to 80% coverage with a 20% co-payment for services [8]. However, co-payments are reduced to 5% or are eliminated for subsidized groups (e.g., households classified as ‘poor’ and ‘near-poor’, children < 6) [4].

By 2020, Vietnam recorded a 91% national SHI coverage rate [7]. Those remaining uninsured mainly consisted of informally employed individuals [7]. Enrollment rates were highest among low- and high-income groups, leaving the so-called “missing middle” of uninsured [5].

Vietnam continues to transition to domestic financing of healthcare from donor financing by expanding the breadth of the national SHI. The Ministry of Health and Vietnam Social Security (VSS) have begun to close service gaps and integrate vertical health programs (e.g., those with stand-alone budget allocations and/or direct donor financing) into SHI financing [7]. The costs for antiretroviral therapy (ART) were transitioned from donor funding to SHI in 2019 [9], COVID-19 treatments were covered by SHI in 2020, and financing for tuberculosis (TB) care was fully transitioned to SHI in 2022 [7].

Until this financing transition, anti-TB medications and consultations were provided free of charge in the public sector, funded by a mixture of domestic and international funding [10]. While first-line TB medications were included in the SHI-reimbursable list of essential medicines, the government network of District TB Units (DTUs) were ineligible for registration with VSS, or reimbursement for services provided. Since July 2022, TB health facilities that met certain conditions could register with VSS and receive reimbursements for TB consultations, diagnostics and anti-TB medications [11]. The financing for drug-resistant (DR-)TB tests and medications remains largely unchanged, co-financed by the Global Fund and domestic budgets [12].

This transition of the TB financing model in Vietnam is a large undertaking as the country has the world’s 10th highest TB burden and the SHI benefits package is already considered to be generous, and the sustainability of the SHI fund is a concern [4, 13] An estimated 169,000 individuals developed TB in 2021, and the disease killed approximately 14,200 [14]. A national costing survey of TB-affected households showed that 63% experienced catastrophic costs, spending ≥ 20% of their annual income on TB [10]. Many face food insecurity and cope with TB-related costs by taking loans, dissavings and informally borrowing money [10, 15, 16].

As Vietnam continues to expand SHI financing for the TB program, it is now vital for people with TB to have SHI. Those without SHI coverage will need to finance their care out of pocket (OOP) or purchase SHI and make co-payments for their care to be subsidized. For these reasons, it is important to understand why certain people with TB are uninsured, the feasibility of enrolling them in insurance when they begin treatment, and the challenges they may face with enrolling in SHI.

## Methods

We conducted a convergent parallel mixed-method case study [17]. A case study was selected because it is well-suited to describe a complex issue in a real-life setting [18]. We used a naturalistic design with theoretical sampling of uninsured persons with TB using an interpretivist approach [19]. Mixed methods were selected to facilitate comparisons between quantitative and qualitative data and interpretation of the findings. An intervention, assisting TB-affected households to enroll in SHI, was conducted between November 2019 and January 2022, prior to the integration of the TB program into the SHI financing scheme. Quantitative data collection sought to answer questions regarding enrollment success rate, time to enrollment and cost of SHI enrollment for uninsured TB-affected households upon TB treatment initiation. The qualitative data explored barriers to SHI enrollment to explain and contextualize the quantitative findings. The quantitative and qualitative data were weighted equally [17].

### Intervention description

A pilot intervention was conducted to facilitate SHI enrollment for people with TB in ten districts of Ha Noi and Ho Chi Minh City (HCMC). The standard process for first-time enrollment into SHI was mapped and costed from a household’s perspective (Additional file 1). Uninsured individuals were identified from the TB treatment register when they were enrolled in drug-susceptible (DS-)TB treatment at DTUs [20]. Study staff then attempted to facilitate enrollment of the person with TB and up to three household members into SHI.

SHI enrollment support included home visits by study staff to provide detailed information and counseling about the process of SHI enrollment, assistance with SHI application preparation including obtaining photocopies of all required documents, follow-up to obtain missing documentation within the household, accompaniment to the SHI office for application submission, and direct payment of the annual SHI premium for the household. For people who did not have the paperwork certifying temporary residence in Hanoi or Ho Chi Minh City, staff visited the local government office to obtain the information about the process for individual cases to obtain residency certificates and support participants with navigation of the bureaucracy. TB-affected people and their household members were also provided with a hotline number to call and receive support during working hours from the social workers who were employed by the study. Study staff attempted to facilitate the SHI enrollment process throughout the entire 6-month duration of DS-TB treatment. After a TB treatment outcome was recorded by the DTU, study staff stopped assisting with SHI enrollment and participants were recorded as ‘not enrolled in SHI’ in the study’s evaluation.

### Quantitative methods

Case-level TB treatment notification data and SHI status were exported from VITIMES, the government-implemented electronic TB register for Vietnam, for all individuals who started TB treatment during the intervention period. The pilot intervention recruited participants from two TB treatment support projects (Project 1, *n* = 59 and Project 2, *n* = 56) [21, 22] and tracked study forms housed in ONA.io. The sample size was determined by the availability of funding provided by the donor for treatment support service delivery, rather than to measure a specific end point of SHI enrollment. Descriptive statistics summarizing the enrollment cascade and turnaround time of enrollment were calculated using Stata v17 (Stata17 Corp, College Station, USA). To obtain the mean costs for household SHI enrollment, total direct costs for purchasing SHI were summed and divided by the total number of participants. Costs were captured in Vietnamese Dong (VND) and converted to United States Dollars (USD) using the exchange rate from the mid-point of the pilot intervention (1 June 2020) from OANDA.com.

### Qualitative methods

Individuals were purposively sampled for maximum variation to ensure representation of all implementation areas and provide gender balance [23].The concept of information power guided the sample size [24]. Given the well-defined study aim, high quality in-depth responses from the participants and the authors’ expertise in the subject area, the sample size of 19 individual interviews and three focus group discussions was deemed appropriate. These were conducted in Ha Noi and HCMC. A total of 34 individuals participated in the interviews (Table 1).

**Table 1:** Participant summary

Participants	Individual interviews (19 total)		Focus group discussions (3 total)		Total Participants	
	Males	Females	Males	Females	Males	Females
Provincial-level TB staff	2	1	–	–	2	1
District-level TB doctors	5	3	–	–	5	3
National TB program staff	1	1	–	–	1	1
Pilot intervention beneficiaries	2	2	6	4	8	6
Community members	–	–	2	3	2	3
Program staff	–	2	–	–	0	**2**
Total participants	19		15		34	

This table has been adapted from the primary analysis of the qualitative interviews, which has been published in Forse et al. [25]

They included 14 people enrolled in the pilot intervention, five community members who were non-beneficiaries of the treatment support intervention, 13 TB program staff from the national-, provincial- and district-levels and two study staff. Interviews were conducted at two time points: June 2019 and 2020. SHI enrollment barriers were collected as part of a qualitative study on the acceptability of providing cash transfers and SHI enrollment to adults with TB [25]. During the second round of interviews in 2020, study staff were included due to their in-depth knowledge of the challenges faced by TB-affected households when attempting to enroll in SHI and their ability to suggest programmatic-level solutions to these challenges. These interviews were conducted one-on-one, after the other interviews and focus groups had been conducted to reduce bias. The interviews were conducted at the National Lung Hospital, HCMC Provincial Lung Hospital, study office or DTUs. All interviews were conducted and transcribed in Vietnamese, translated into English, checked and finalized by a lead translator.

The interviews were analyzed through an inductive approach and themes were drawn through a framework analysis [26] to identify barriers to enrolling in SHI using Dedoose Version 7.0.23 (SocioCultural Research Consultants, Los Angeles, USA).

### Data triangulation

Quantitative and qualitative data were collected in parallel. Triangulation of quantitative and qualitative data was conducted to synthesize findings and assess the level of agreement, convergence, and divergence from the findings generated by the different methods [17].

## Results

During the study, 5887 individuals were treated for DS-TB across the 10 intervention districts (Table 2). TB registers indicated that 2846 (48.3%) individuals were uninsured upon treatment initiation, or their SHI enrollment status was not recorded. Among 115 uninsured study participants, 88 (76.5%) were successfully enrolled in SHI before the end of their TB treatment. Among those, the household had an average of two members, resulting in a total of 206 individuals living in TB-affected households receiving SHI coverage through the pilot intervention.

**Table 2:** Descriptive Statistics on Social Health Insurance (SHI) Enrollment

Indicator/variable	Value
Number of people treated for drug susceptible (DS-)TB during the pilot intervention	5887
Number of people unknown or without SHI at treatment initiation (%)	2846 (48.3%)
**Number of participants in the pilot intervention (%)**	**115 (4.0%)**
**Number of participants with DS-TB enrolled in SHI (%)**	**88 (76.5%)**
Median (IQR) days between treatment start and SHI card issuance	34.5 (24–68)
Median (IQR) days for study enrollment	11 (5–23)
Median (IQR) days for SHI application submission	7 (1–19.5)
Median (IQR) days for SHI application processing	12 (9–20)
Average household members included in SHI enrollment	2.3
Total household members included in SHI enrollment	206
Mean direct cost for household SHI enrollment (VND^1^)	1,503,313
Mean direct cost for household SHI enrollment (USD^2^)	65.52
**Number of participants not enrolled in SHI within treatment duration**	**27 (23.5%)**
Due to missing documents essential for enrolment (%)	19 (70.4%)
Due to refusal from family member to enroll for household (%)	4 (14.8%)
Due to death of participant before SHI enrollment (%)	1 (3.7%)
Other reasons (%)	3 (11.1%)

The bold values are headings in the SHI enrollment cascade. The values underneath them (not in bold) are a subset of the value in bold

^1^VND: Vietnamese Dong

^2^USD: United States Dollar

The median time between DS-TB treatment initiation and SHI card issuance was 34.5 days (IQR 24–68): 11 days (IQR 5–23) between treatment initiation and pilot enrollment, 7 days (IQR 1–19.5) for SHI application preparation and submission, and 12 days (IQR 9–20) for application processing and SHI card provision.

The qualitative data showed that participants across all participant groups broadly understood that SHI is a system designed to prevent catastrophic OOP medical expenditure. As shown in Table 3, National and provincial-level TB staff described SHI as a human right and spoke about achieving UHC as a nation; no other participant groups discussed SHI in this way. However, district-level doctors and intervention beneficiaries spoke in greater details about coverage and service gaps, and the practicalities of utilizing SHI. These participant groups expressed that when individuals purchase SHI only after a negative health event, such as a TB diagnosis, then the social safety net is unavailable to provide support until SHI coverage begins. Drawn from these views, the first theme indicated that the optimal time to purchase SHI is prior to a TB diagnosis.

**Table 3:** Contributions to themes among participant types

	Theme	Participant groups providing statements supporting the theme
1	The optimal time to purchase SHI is prior to a TB diagnosis	District-level TB doctors Pilot intervention beneficiaries
2	Perceived lack of knowledge about SHI enrollment procedures prevents or delays enrollment	District-level TB doctors Program staff
3	SHI enrollment costs were perceived as prohibitively high for some	District-level TB doctors Provincial-level TB staff Pilot intervention beneficiaries Community members
4	Some individuals do not possess the required documentation to obtain SHI	District-level TB doctors Pilot intervention beneficiaries Program staff
5	Current SHI enrollment procedures may prevent full population coverage	Provincial-level TB staff District-level TB doctors Community members Program staff

One DTU staff member described how the standard processing time, or delays in processing SHI applications led to periods of high OOP expenditure:

“Unfortunately, claims are not immediately paid upon [SHI registration] submission. They may be handled in about 2 or 3 weeks, or even one month. That is why the insurance is not available at the time that they want to go for an examination and treat their condition using insurance.” (Female, District-level TB staff)

A complementary theme was that perceived lack of knowledge about SHI enrollment procedures prevents or delays enrollment. District-level TB doctors and program staff identified a lack of understanding and knowledge of the SHI enrollment process as a main contributor to lack of insurance or delays in obtaining coverage.

“Actually, for some people [with TB] who do not clearly understand the [enrollment] procedures… it will take a lot of time [to obtain SHI]. It also depends on the staff who handle the files at the commune; some staff are very enthusiastic and they help patients complete forms. There are cases [...] where they [people with TB] are required to fill in all information and write specific codes of each insurance card [from other family members] on a form. Meanwhile some people in their family work far from home and cannot send their insurance cards home in a timely manner.” (Female, program staff)

Participants tended to believe that individuals who lacked information about SHI made up the small minority of uninsured people in Vietnamese society. The above quote illustrated that the complicated administrative process prohibits enrollment; however, a factor potentially facilitating SHI enrollment may be the helpfulness of the person processing the SHI application.

The average cost per household to obtain SHI enrollment for one year (Table 2) was VND 1,503,313 (USD 65.52). (For detailed information on the costs of SHI enrollment, see Additional file 1). A third theme contextualized this finding and showed that SHI enrollment costs were perceived as prohibitively high for some. Cost was a greater challenge for lower income families, who did not meet the government’s criterion of households classified as ‘poor’ or ‘near-poor’, and were therefore ineligible for premium subsidies and SHI registration with lower co-payment rates. One DTU doctor reported that:

“We think that it is simple to buy health insurance cards, but that is only true for those who have sustainable income - when our income is much higher than the fee for buying health insurance. For some people, buying health insurance is a luxury.” (Male, District-level TB staff)

Twenty-seven people with TB (23.5%) were unable to obtain SHI coverage. The primary reason (70.4%) was missing documentation. In four instances (14.8%) a household member other than the person with TB refused to enroll in SHI. One individual (3.7%) died during the enrollment process. Three individuals (11.1%) did not enroll for other reasons.

SHI refusal by household members was not identified as a barrier to SHI enrollment in the qualitative data. However, a fourth theme confirmed the primary reason for non-enrollment by showing that some individuals do not possess the required documentation to obtain SHI, such as their identity card or ‘family book.’ [See Supplementary File] Even with six months of support from study staff, some TB-affected households were unable to gather the required documents for enrollment. The following quotation by an undocumented, elderly woman with TB illustrates the prolonged challenges she faced with obtaining formal employment, access to government services and SHI:

“I have had problems with my personal papers for a few decades and I cannot adjust my papers because I don’t have the money. […] I searched for my Identity Card and found out that I had lost it. Then I came back there [my hometown] to get the family book, to reissue my ID and to get my CV certified so I could join a company. I was very young at that time, just a little bit more than thirty years old, and I learned that I was cut from the family book.” (Female, pilot beneficiary)

To address challenges with documentation, one DTU officer in HCMC suggested that individuals who had never been insured required a change to the SHI registration requirements to ensure that everyone in Vietnam can access SHI:

“I think we should be flexible with these cases or we can find another way. Normally, the people who really need the support and the insurance or cash support, they are the people who have less information. […] We cannot have the same requirements for these people as for other people. Actually, for those who have [met] all conditions, they already have health insurance cards.” (Male, District-level TB staff)

Participants expressed that the uninsured had often not purchased SHI for a reason, and alternative registration procedures were needed to make SHI accessible for all. A fifth theme was identified indicating that current SHI enrollment procedures may prevent full population coverage.

Beyond the undocumented, some participants reported the enrollment mandate for the entire household (made under the Amendment to the HIL) for first-time enrollees was viewed as prohibitive of SHI coverage.

“Because in the old days, health insurance was sold individually for each person, but now it is sold to households, and many households do not have as good economic [situation]… so they can only afford to buy it for 50% or 60% of the household. Unskilled labor or low-income labor cannot afford to buy it for the whole family. That is to say, it is easier to buy it for each individual and it is difficult to buy for the whole family.” (Male, community member)

Though individual registration would make SHI more accessible to individuals with TB due to lower annual costs, household members with high vulnerability to TB would not be covered if policy promoted individual enrollment solely for TB.

## Discussion

This mixed-methods case study showed that by providing full subsidy and registration assistance, most uninsured people with TB could access SHI. However, the median time to insurance coverage meant that approximately 20% of a person’s DS-TB treatment duration remained uncovered by SHI despite successful enrollment. A substantial number of participants were unable to enroll in SHI and are likely to be perpetually locked out of SHI due to lack of personal documentation. Additional barriers to SHI enrollment were found to be lack of knowledge, the cost of obtaining coverage, and the household-based registration requirement.

The pilot intervention had dedicated staff who facilitated SHI application development and submission, yet it still took a median of 34.5 days for SHI coverage to take effect. In a context where this level of support is not available to all people with TB, it is likely that the turnaround time for SHI coverage is longer due to the complicated bureaucracy involved. This poses a major challenge, as TB-affected households incur the highest cost during the first two months of treatment [15]. One cost avoidance/mitigation strategy that people with a TB diagnosis may employ following the health financing transition is delaying TB treatment initiation until SHI coverage commences. This will likely lead to worse outcomes and sustained community transmission. The time between diagnosis and treatment should be rigorously monitored to ensure that this coping strategy is not employed, and alternative support should be made available to ensure that people diagnosed with TB are able to receive immediate treatment.

With the TB health financing transition, the uninsured will be asked to pay OOP for TB treatment and most insured individuals must co-pay for TB services which were previously provided free of cost. A national patient cost survey in 2018 found that 63% of TB-affected households experienced catastrophic costs under the previous health financing model [10]. There is a risk that the proportion of TB-affected households experiencing catastrophic costs could increase with the introduction of fees. This was not found to be the case for people living with HIV (PLHIV) when the costs of ART transitioned to SHI in Vietnam, but a new nationally representative TB costing survey is needed to assess this risk [9]. Several domestic solutions could ameliorate these challenges. As suggested for the Indian context, domestic revenues allocated by the Ministry of Finance to VSS could be increased to better support TB care [27]. VSS could also reclassify the category of TB disease and thus ensure that SHI paid for all diagnostics and drugs associated with TB treatment, without the need for a co-payment. A mid-term review of the Global Fund program in Vietnam has also called for a SHI package specifically designed to cover the OOP medical costs of TB care [28]. There are several potential mechanisms to prevent costs from falling on TB-affected households. A deeper investigation is needed to understand the fiscal space available within the Vietnamese government to cover such costs.

This case study showed that 23.5% of the uninsured people with TB were never able to enroll for the duration of their treatment, primarily due to lack of documentation. Specific provisions need to be made for the undocumented to receive free TB diagnosis, consultations, and medications through routine practice of the TB program. Multi- and bi-lateral funding mechanisms can also play a role in filling gaps by paying for TB tests for the uninsured, purchasing SHI for those diagnosed with TB, subsidizing or reimbursing OOP expenditure in the period before SHI coverage takes effect, and fully financing TB care for the undocumented. Furthermore, longer-term health system strengthening initiatives, such as creating a legal mechanism for the undocumented to obtain SHI, are likely needed to address the challenges faced by the 9% of the general population that remain uninsured. The ILO has called for “determining new strategies, which may include extension of state budget-funded subsidies to further support the participation of workers in the informal economy [7].” These forms of inclusive initiatives would solve the TB-specific challenges identified in this study and have a large positive impact on society.

We found that addressing the cost of SHI premiums and knowledge gaps in the enrollment procedures may improve SHI coverage. These findings mirror those following the transition of HIV financing to SHI in 2017. A study among PLHIV identified burdensome processes, lack of information about SHI registration procedures, and high SHI premium costs for a household as key barriers to SHI coverage [29]. However, a cluster randomized control trial which provided education, a 25% premium subsidy, or both to uninsured households found that these interventions had limited effects on SHI enrollment. Yet, “less healthy” individuals had higher SHI enrollment rates [30]. This suggests that people who have just received a TB diagnosis could be more receptive to interventions promoting SHI enrollment through premium subsidization and education. Vietnam’s National TB Program (NTP) has established a fund to subsidize SHI enrollment costs for TB-affected individuals. The size of the fund could be increased with additional support while access to the fund and the procedures for receiving support could be optimized [31]. Given the SHI transition, the NTP should also consider providing educational materials about the SHI enrollment process through the DTU network to uninsured persons with TB.

TB registers indicated that 52% of people starting TB treatment in the urban intervention districts had recorded SHI coverage. This rate is lower than other recent SHI coverage reports. A 2018–2022 DS-TB costing survey reported a SHI coverage of 70% [32], while in a DR-TB costing survey (2020–2022) it was 85% [16]. All available data sources indicate that SHI coverage among people with TB is lower than the general population, which is indicative of their socioeconomic vulnerability [33]. However, this large SHI coverage rate discrepancy may be explained by people with TB not revealing they had SHI coverage, or DTU staff could have also inconsistently recorded an individual’s SHI status in the paper TB registers since these data did not have much clinical relevance for TB treatment at the time. Now that DTUs receive financial reimbursements for the TB services from VSS, SHI coverage rates in treatment registers are likely to increase. Further research should be conducted to understand the national SHI coverage rate for people receiving TB treatment, along with the risk factors associated with being uninsured.

### Limitations

This case study was conducted in the two largest cities of Vietnam and findings may not be representative of the entire country. Quantitative data were collected in a programmatic setting, and SHI coverage data for all individuals initiating TB treatment in the intervention areas appear to be underreported for reasons described above. Lastly, we were unable to collect SHI enrollment data from a control population, either prospectively during the pilot intervention or retrospectively during the pilot evaluation. As a result, we do not have information on the enrollment status or time to obtain SHI coverage among a population that did not receive assistance from the pilot intervention. However, given the substantial additional support provided by study staff for the enrollment process, we believe it is safe to assume that if left alone, TB-affected households would be slower in the enrollment process and likely enroll in lower rates.

## Conclusion

Vietnam is viewed as a leader among Southeast Asian nations in its commitment and progress towards UHC. This mixed-methods case study illustrated the progress that Vietnam has made in its path to greater domestic financing of healthcare through SHI. This study is one of the first to examine the integration of TB services into SHI in Vietnam and define the challenges that people with TB face while attempting to gain access to financial protection after receiving a TB diagnosis. In order to make strides towards UHC in Vietnam and to close population coverage gaps, initiatives are required to specifically address the barriers faced by the uninsured. This study found that the majority of the uninsured were able to gain access to SHI through full subsidization of premiums, enrollment assistance and education. However, initiating TB care and SHI enrollment concomitantly left a significant portion of the 6-month TB treatment duration without financial protection. Additionally, a quarter of the uninsured with TB were unable to gain access to SHI during treatment, primarily due to a lack of documentation. There is great need for official mechanisms to be in place that enable those without sufficient state documents to access the TB program and to address the time-sensitive nature of providing effective financial protection during treatment of an infectious disease. These findings are relevant for other high TB burden, middle-income countries who are on a similar pathway for transitioning away from donor-financed TB programs to ones supported with a higher proportion of domestic resources.

### Supplementary Information

**Additional file 1.** Mapping of procedures and costs for first-time enrollment into Vietnam's social health insurance scheme.

## Data Availability

The quantitative dataset used and analyzed during the current study are available from the corresponding author on reasonable request. Seven anonymized transcripts of interviews with the people enrolled in the pilot intervention and non-beneficiaries have been uploaded to the following URL: https://doi.org/10.5281/zenodo.7736220.

## Abbreviations

ART: Anti antiretroviral therapy
DR-TB: Drug resistant tuberculosis
DS-TB: Drug susceptible tuberculosis
DTU: District TB Unit
HCMC: Ho Chi Minh City
HIL: Health Insurance Law
HIV: Human immunodeficiency virus
ILO: International Labour Organization
IQR: Interquartile range
NTP: National Tuberculosis Program
OOP: Out of pocket
PLHIV: People Living with HIV
SHI: Social Health Insurance
TB: Tuberculosis
UHC: Universal Health Coverage
USD: United States Dollar
VND: Vietnamese Dong
VSS: Vietnam Social Security
