# Supplementary material for: Towards universal health coverage in Vietnam: a mixed-method case study of enrolling people with tuberculosis into social health insurance
Source: Health Res Policy Syst. 2024 Apr 2;22:40. doi: 10.1186/s12961-024-01132-8 (PMC10985876; doi:10.1186/s12961-024-01132-8)
Supplement: Supplementary file 1 — Additional file 1. Mapping of procedures and costs for first-time enrollment into Vietnam's social health insurance scheme. [file 12961_2024_1132_MOESM1_ESM.docx]

The process for first-time Social Health Insurance (SHI) enrollment for a household was mapped and was found to have three steps:

**Step 1:** The household needs to obtain original versions and copies of the either the family book (*so ho khau-* family book, a national system of family registry and address registration (1)) or temporary residence book (*KT3*) or long-term resident form (*HK09*) at the current residence; the original identification (ID) card of the person who is registering for SHI; and a copy of the ID cards and SHI cards of the family members who already have SHI cards in the household (2).

**Step 2:** This documentation, a registration form (TK0-TS), and the annual insurance fee for the entire household would be submitted to the SHI agency (3). Vietnam Social Security (VSS) then would process the documentation. A national guideline (Decision No. 595/QD-BHXH) indicates that the processing time required to issue the SHI card is five days from the date of submission (4).

**Step 3:** Issuance of SHI card. The individual visits the SHI agency where payment was submitted and collects the card. For the majority of registration groups, SHI benefits for the individual become active upon receipt of the card. However, for household members (along with sub-groups such as near-poor, school pupils, and university students) SHI benefits are activated for use 30 days after the date of payment (5).


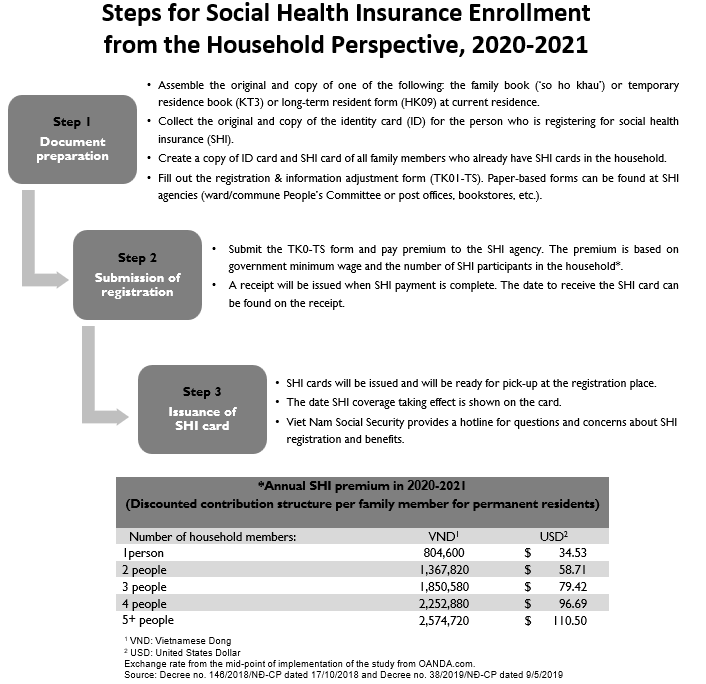


**References:**

1. Hardy A. Rules and Resources: Negotiating the Household Registration System in Vietnam under Reform. Sojourn: Journal of Social Issues in Southeast Asia [Internet]. 2001 [cited 2023 May 30];16(2):187–212. Available from: https://www.jstor.org/stable/41057062

2. Law No. 81/2006/QH11 of November 29, 2006, on residence [Internet]. Sect. Law No. 81/2006/QH11 of November 29, 2006, on residence, 81/2006/QH11 Nov 29, 2006. Available from: https://vanbanphapluat.co/law-no-81-2006-qh11-of-november-29-2006-on-residence

3. Thủ tục hành chính: Đăng ký đóng, cấp thẻ BHYT đối với người chỉ tham gia BHYT [Details of Small Administrative Procedures: Register to pay and issue health insurance cards for people only participating in health insurance] [Internet]. Bao Hiem Xa Hoi [Vietnam Social Security]. [cited 2022 Aug 26]. Available from: https://baohiemxahoi.gov.vn:443/thu-tuc-hanh-chinh/Pages/default.aspx?ItemID=73

4. Viet Nam Social Security. Quyết định số 595/QĐ-BHXH ngày 14 tháng 4 năm 2017 của Tổng giám đốc bảo hiểm xã hội Việt Nam về việc ban hành quy trình thu bảo hiểm xã hội, bảo hiểm y tế, bảo hiểm thất nghiệp, bảo hiểm tai nạn lao động - bệnh nghề nghiệp, quản lý sổ bảo hiểm xã hội, thẻ bảo hiểm y tế [Decision No: 595/2017/QD-BHXH dated April 14, 2017 of the Director General of Vietnam Social Security on promulgating the process of collecting social insurance, health insurance, unemployment insurance, accident insurance labor - occupational diseases, management of social insurance books, health insurance cards] [Internet]. Decision No: 595/2017/QD-BHXH Bảo hiểm Xã hội Việt Nam; Apr 14, 2017. Available from: http://vanban.bhxhtphcm.gov.vn/handle/BHXHTPHCM_123456789/78

5. BAN HÀNH QUY TRÌNH THU BẢO HIỂM XÃ HỘI, BẢO HIỂM Y TẾ, BẢO HIỂM THẤT NGHIỆP, BẢO HIỂM TAI NẠN LAO ĐỘNG, BỆNH NGHỀ NGHIỆP; QUẢN LÝ SỔ BẢO HIỂM XÃ HỘI, THẺ BẢO HIỂM Y TẾ [Procedures for the Collection of Social Insurance, Health Insurance, Unemployment Insurance, Labor Accident Insurance, Occupational Disease; Management of Social Insurance Books, Health Insurance Card.] [Internet]. 2089/VBHN-BHXH Jun 26, 2020. Available from: https://thuvienphapluat.vn/van-ban/Bao-hiem/Van-ban-hop-nhat-2089-VBHN-BHXH-2020-Quy-trinh-thu-bao-hiem-xa-hoi-bao-hiem-y-te-454887.aspx
